# Supplementary figures and images for: MiR-106a-5p by Targeting MAP3K2 Promotes Repair of Oxidative Stress Damage to the Intestinal Barrier in Prelaying Ducks
Source: Animals (Basel). 2024 Mar 28;14(7):1037. doi: 10.3390/ani14071037 (PMC11010895; doi:10.3390/ani14071037)

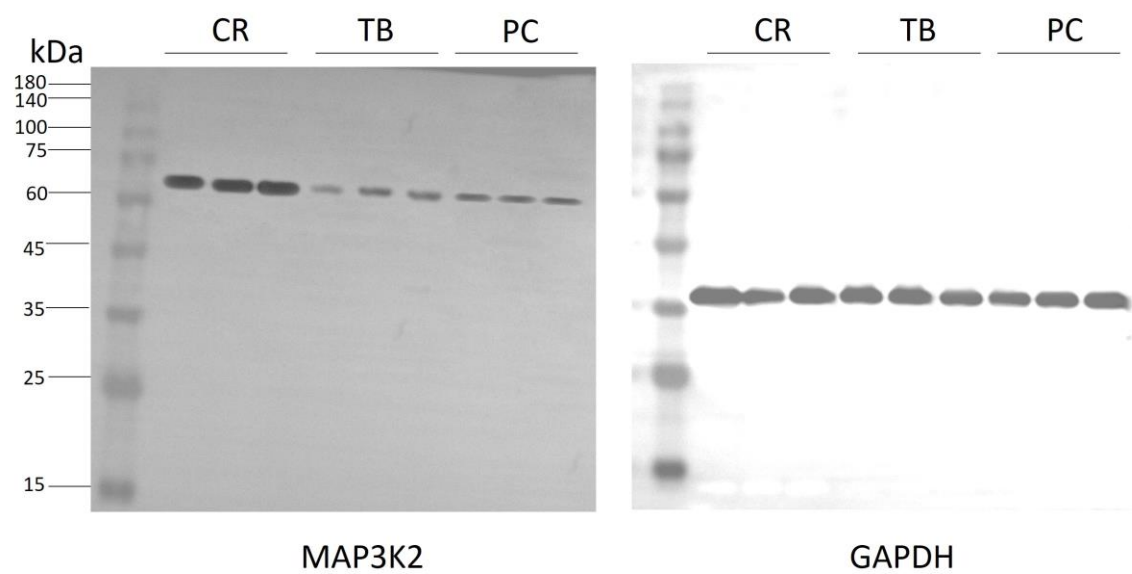

Figure S1: Original Western Blot figure of Figure 8(C).

Supplement: Supplementary file 1 [file animals-14-01037-s001.zip › animals-2859081-supplementary.pdf]
